# QT Interval Prolongation with One or More QT-Prolonging Agents Used as Part of a Multidrug Regimen for Rifampicin-Resistant Tuberculosis Treatment: Findings from Two Pediatric Studies

**DOI:** 10.1128/aac.01448-22

**Published:** 2023-06-26

**Authors:** Ali Mohamed Ali, Kendra K. Radtke, Anneke C. Hesseling, Jana Winckler, H. Simon Schaaf, Heather R. Draper, Belén P. Solans, Louvina van der Laan, Jennifer Hughes, Barend Fourie, James Nielsen, Anthony J. Garcia-Prats, Rada M. Savic

**Affiliations:** a Department of Bioengineering and Therapeutic Sciences, University of California San Francisco, San Francisco, California, USA; b Bagamoyo Research and Training Center, Ifakara Health Institute, Bagamoyo, Tanzania; c Desmond Tutu TB Centre, Department of Paediatrics and Child Health, Faculty of Medicine and Health Sciences, Stellenbosch University, Stellenbosch, South Africa; d Department of Pediatrics, New York University School of Medicine, New York, New York, USA; e Department of Pediatrics, University of Wisconsin-Madison School of Medicine and Public Health, Madison, Wisconsin, USA

**Keywords:** drug-drug interaction, tuberculosis, pediatrics, QT prolongation

## Abstract

Rifampicin-resistant tuberculosis (RR-TB) involves treatment with many drugs that can prolong the QT interval; this risk may increase when multiple QT-prolonging drugs are used together. We assessed QT interval prolongation in children with RR-TB receiving one or more QT-prolonging drugs. Data were obtained from two prospective observational studies in Cape Town, South Africa. Electrocardiograms were performed before and after drug administration of clofazimine (CFZ), levofloxacin (LFX), moxifloxacin (MFX), bedaquiline (BDQ), and delamanid. The change in Fridericia-corrected QT (QTcF) was modeled. Drug and other covariate effects were quantified. A total of 88 children with a median (2.5th-to-97.5th range) age of 3.9 (0.5 to 15.7) years were included, of whom 55 (62.5%) were under 5 years of age. A QTcF interval of >450 ms was observed in 7 patient-visits: regimens were CFZ+MFX (*n* = 3), CFZ+BDQ+LFX (*n* = 2), CFZ alone (*n* = 1), and MFX alone (*n* = 1). There were no events with a QTcF interval of >500 ms. In a multivariate analysis, CFZ+MFX was associated with a 13.0-ms increase in change in QTcF (*P* < 0.001) and in maximum QTcF (*P* = 0.0166) compared to those when other MFX- or LFX-based regimens were used. In conclusion, we found a low risk of QTcF interval prolongation in children with RR-TB who received at least one QT-prolonging drug. Greater increases in maximum QTcF and ΔQTcF were observed when MFX and CFZ were used together. Future studies characterizing exposure-QTcF responses in children will be helpful to ensure safety with higher doses if required for effective treatment of RR-TB.

## INTRODUCTION

Multidrug-resistant (MDR) and rifampicin-resistant (RR) tuberculosis (TB) represents a major threat to the fight against TB globally. In 2020 alone, there were 132,222 bacteriologically confirmed cases of MDR/RR-TB, which is less than a third of the total estimated MDR/RR-TB cases (1). MDR/RR-TB affects people of all age groups, including children less than 15 years old (2), who account for 25,000 to 32,000 new MDR/RR-TB cases annually (3, 4).

RR-TB is typically treated with 4 to 7 drugs for a duration of 9 to 24 months (5). Treatment guidelines have undergone major revision in the last decade, moving from injectable-based regimens to all-oral regimens including new or repurposed drugs. All-oral regimens have demonstrated excellent efficacy and potential for treatment shortening in adults, and their use has been recently extended to children. However, safety is an important consideration for use of these drugs. Several of the recommended second-line and novel TB drugs are associated with QT interval prolongation, including moxifloxacin (MFX), bedaquiline (BDQ), delamanid (DLM), and clofazimine (CFZ) (6–11). An absolute corrected QT measured at >500 ms is considered to increase the risk for potentially fatal arrhythmias (12–14). RR-TB treatment regimens are mostly composed of combinations of at least two of these agents together (5). Concomitant use of more than one QT interval-prolonging drug increases the risk of QT-related adverse events (15–21). However, the associated risk of QT interval prolongation among second-line drugs recommended for RR-TB has not been well characterized in children, especially when drugs are used in combination.

The objective of this study was to characterize QT interval prolongation in children (aged 0 to 17 years) routinely treated for RR-TB who received at least one QT-prolonging drug such as BDQ, MFX (9, 10), and CFZ (22–24).

## RESULTS

### Study participants.

A total of 88 study participants treated for RR-TB with one or more QT interval-prolonging drugs were included in this analysis. Baseline characteristics of participants are shown in Table 1. The median (2.5th-to-97.5th range) age was 3.9 (0.5 to 15.7) years. Fifty-five (62.5%) children were under 5 years of age. Eight children (9.1%) were HIV positive. There were 32 (36.4%) children receiving MFX+CFZ and 16 (18.2%) children receiving BDQ+CFZ. No child received MFX+BDQ. The median (2.5th-to-97.5th range) dose at sampling was 11.8 (8.9 to 16.9) mg/kg of body weight daily for MFX, 31 (13 to 47) mg/kg weekly for CFZ, and 12.1 (5.9 to 75.3) mg/kg weekly for BDQ. Electrocardiogram (ECG) sampling in 6/19 (30%) children on BDQ occurred during daily dosing (loading phase); the remainder were in the maintenance phase (thrice-weekly dosing). Children in the CFZ, CFZ+MFX, and CFZ+BDQ groups had received CFZ for a mean 46.5, 71, and 68.5 days prior to ECGs, respectively (see Table S1 in the supplemental material).

**TABLE 1:** Baseline characteristics of study participants^*b*^

Characteristic	Value
*N*	88
Female sex, *n* (%)	47 (53.4)
Any use of drug^*a*^:	
LFX	63 (71.6)
CFZ	62 (70.5)
MFX	59 (67.0)
Only monotherapy with drug:	
LFX	16 (18.2)
CFZ	7 (8.0)
MFX	26 (29.5)
Drug combination^*a*^	
CFZ+LFX	37 (42.0)
CFZ+MFX	32 (36.4)
CFZ+BDQ	4 (4.5)
CFZ+DLM	3 (3.4)
LFX+BDQ	1 (1.1)
LFX+DLM	1 (1.1)
CFZ+LFX+BDQ	11 (12.5)
CFZ+LFX+DLM	2 (2.3)
CFZ+MFX+DLM	1 (1.1)
CFZ+BDQ+DLM	1 (1.1)
CFZ+LFX+BDQ+DLM	2 (2.3)
HIV positive, *n* (%)	8 (9.1)
Age (yr)	3.9 (0.5, 15.7)
Wt (kg)	13.8 (6.6, 51.6)
Ht (cm)	96.2 (64.7, 164.3)
Underweight	4/55 (7.3)
Stunted	12/55 (21.8)
Wasted	3/55 (5.5)

a Patient could have received more than one drug and hence could be counted more than once. Some children switched between LFX and MFX as part of the study design. CFZ, clofazimine; MFX, moxifloxacin; LFX, levofloxacin; BDQ, bedaquiline; DLM, delamanid.

b Antituberculosis drugs refer only to those with QT-prolonging effects. Values are reported as *n* (%) or median (2.5th, 97.5th percentiles); underweight, weight-for-age z-score of <−2; stunted, height-for-age z-score of <−2; wasted, weight-for-height z-score of <−2. A patient was considered to receive a particular drug if the patient received it at the time of ECG.

### QTcF interval prolongation.

**FIG 1:**
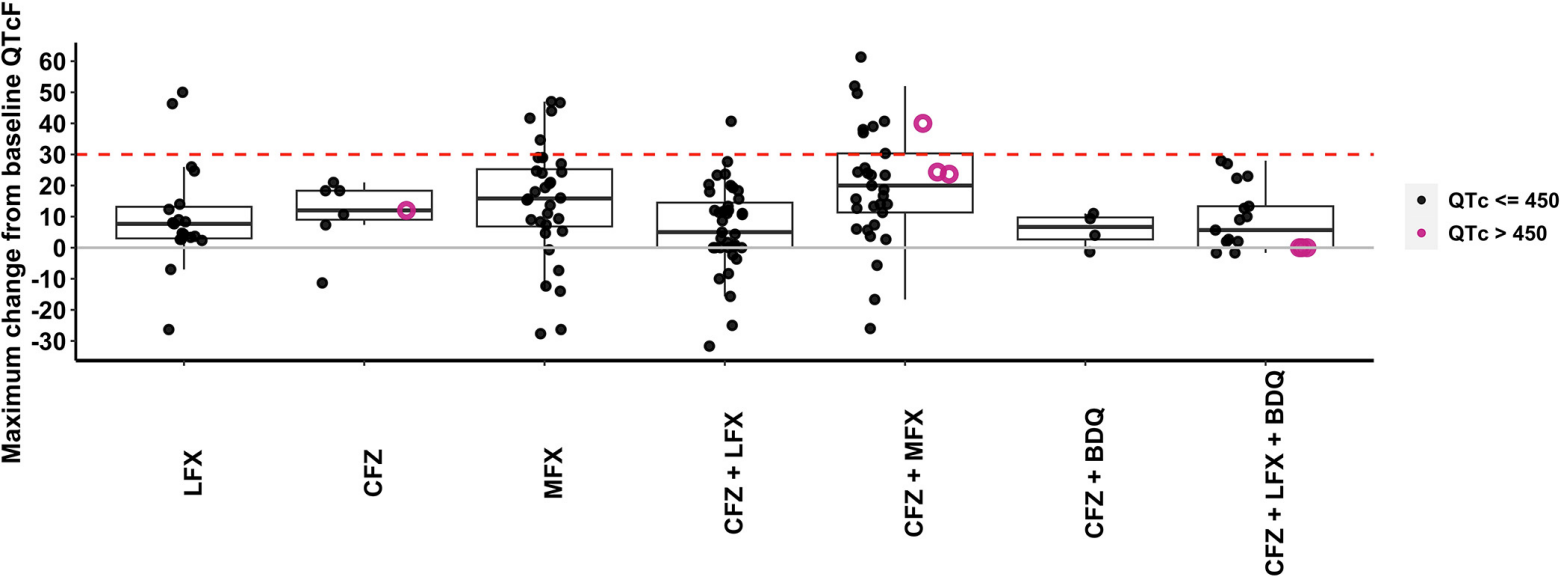
Maximum change in QTcF (milliseconds) after drug administration by drug regimen. LFX, levofloxacin; CFZ, clofazimine; MFX, moxifloxacin; BDQ, bedaquiline. A child can appear more than once and on more than one drug regimen depending on visit. Solid circles are observations where absolute QTcF is less than or equal to 450 ms. Open circles are observations where absolute QTcF is greater than 450 ms. The dashed red line represents a change in QTcF from predose of 30 ms. The solid gray line represents a change in QTcF from predose of 0 ms. QTcF, QT interval corrected by Fridericia formula.

The number of QT prolongation events is shown in Table 2. There were 442 ECG measurements collected after drug administration in 159 patient-visits. Sixty-three patients had ECG measurement on visit 1, while 50 patients had ECG measurement on visit 2 and only one patient had ECG measurement on visit 3. No grade 3 (severe) Fridericia-corrected QT (QTcF) adverse events occurred with any drug regimen. Five of the seven grade 1 QTcF adverse events occurred in children taking two or more QT-prolonging drugs. Study participants who received CFZ+MFX had the greatest maximum ΔQTcF (20.0 ms), followed by those receiving MFX alone (15.8 ms) and CFZ alone (12.0 ms) (Table 2; Fig. 1). Of the 18 patient-visits with a maximum ΔQTcF of >30 ms (Table 2), 6 of these occurred at 1 h, 2 at 2.5 h, 4 at 4 h, and 5 at 10 h after drug administration. One patient (HIV-negative female aged 12 years) had a ΔQTcF of 61.0 at 4 h and 61.3 ms at 10 h after drug administration.

**TABLE 2:** Summary of QTcF prolongation by drug regimen^*b*^

Parameter	Value for drug or combination:							
	LFX alone	CFZ alone	MFX alone	CFZ+MFX	CFZ+LFX	CFZ+BDQ	CFZ+LFX+BDQ	CFZ+LFX+DLM
Total no. of patient-visits^*a*^	19	7	32	33	39	4	16	5
No. of QT prolongation events (QTcF of >450 ms)	0	1	1	2	0	0	3	0
Grade 1 (mild)	0	1	1	2	0	0	3	0
Grade 2 (moderate)	0	0	0	0	0	0	0	0
Grade 3 (severe)	0	0	0	0	0	0	0	0
No. of events with ΔQTcF of >30 ms	2	0	5	9	1	0	0	0
No. of events with ΔQTcF of >60 ms	0	0	0	1	0	0	0	0
Maximum QTcF, ms	397 (351, 442)	393 (368, 491)	408 (366, 464)	420 (380, 482)	407 (343, 446)	417 (371, 446)	426 (352, 473)	409 (403, 440)
Maximum ΔQTcF, ms	7.7 (−26.3, 50.0)	12.0 (−11.3, 21.0)	15.8 (−27.7, 47.0)	20.0 (−26.0, 61.3)	5.0 (−31.7, 40.7)	6.7 (−1.3, 11.0)	5.7 (−75.3, 28.0)	24.7 (8.3, 27.0)
Time after dose at maximum QTcF, h	1.4 (0, 10.5)	1.2 (0, 4.6)	3.6 (0, 10.4)	3.6 (0, 10.3)	1.0 (0, 9.6)	0.1 (0, 10.2)	0 (0, 10.2)	4.2 (1.5, 10.4)

a A patient can appear multiple times due to multiple visits and can be represented in multiple treatment groups if the regimen changed between visits. Values are reported as *n* or median (2.5th, 97.5th percentile range).

b Abbreviations: LFX, levofloxacin; CFZ, clofazimine; MFX, moxifloxacin; BDQ, bedaquiline; DLM, delamanid; QTcF, QT interval corrected by Fridericia formula; ΔQTcF, change in QTcF from time zero; mild QT prolongation, QTcF interval of >450 to ≤480 ms; moderate QT prolongation, QTcF interval of >480 to ≤500 ms; severe QT prolongation, QTcF interval of >500 ms. The maximum QTcF was calculated using individual QTcF measures, and the grading was done with the mean of the triplicate.

### Risk factors for QTcF prolongation.

Age, predose QTcF value (QTcF_0_), and CFZ+MFX use were significantly associated with maximum ΔQTcF in multivariate linear regression (*P* < 0.01) (Table S2). The maximum ΔQTcF was 13.4 ms greater in CFZ+MFX recipients.

Analysis of all ΔQTcF values after drug administration found significant associations with CFZ, MFX, CFZ+LFX (levofloxacin), CFZ+MFX, and CFZ+BDQ as well as MFX dose, LFX dose, age, and weight in univariate analysis (Table S3). DLM could not be tested due to a small sample size (*n* = 7). Table 3 presents the multivariate analysis results of ΔQTcF using linear mixed-effects modeling. The typical ΔQTcF among all participants was 3.7 ms. There was no significant difference in the typical ΔQTcF between participants receiving LFX and participants receiving MFX- or CFZ-based regimens without LFX (ΔOFV [objective function value] = −3.39, *P* = 0.0654). Each 10-ms increase in QTcF_0_ was associated with a 3.4-ms decrease in ΔQTcF, and a 1-year increase in age was associated with a 1.3-ms increase in ΔQTcF. The combination of CFZ and MFX (CFZ+MFX) was significantly associated with a 13.0-ms (3.5-fold) increase in ΔQTcF (ΔOFV = −23.51, *P* < 0.001). No other covariates from the univariate analysis were significant in multivariate analysis, including time after dose.

**TABLE 3:** Final model of the change in QTcF after dose^*a*^

Parameter	Value (% RSE)
Typical ΔQTcF in all participants, θ_TYP_ (ms)	3.7 (44.6)
Effect of taking both clofazimine and moxifloxacin on ΔQTcF, θ_CFZ+MFX_ (ms)	13.0 (55.4)
Effect of age on ΔQTcF, θ_age_ (ms/yr)	1.3 (24.6)
Effect of predose QTcF on ΔQTcF, $\theta_{{\text{QTcF}}_{0}}$ (ms/10 ms)	−3.4 (14.0)
Between-subject variability, η_BSV_ (ms)	7.2 (20.8)
Between-occasion variability, η_BOV_ (ms)	9.2 (12.8)
Residual additive error, ε (ms)	11.1 (4.2)

a Abbreviations: RSE, relative standard error; ΔQTcF, change in QTcF (ms) after drug administration; QTcF, QT interval corrected by Fridericia formula; ΔQTcF values after drug administration for levofloxacin-based regimens (ΔQTcF_LFX_) and moxifloxacin- or clofazimine-based regimens (ΔQTcF_MFX/CFZ_) were described as a function of age (θ_age_), predose QTcF (i.e., QTcF at time zero [QTcF_0_]), and concomitant drug use as described below: ${\Delta \text{QTcF}}_{\text{LFX}}\;\text{=}\;\theta_{\text{TYP}}\;+\;\theta_{\text{age}}\;\times \;\left(\text{age}-2.83\right)\;+\;\theta_{{\text{QTcF}}_{\text{0}}}\;\times \;\left(\frac{{\text{QTcF}}_{0}-363}{\text{10}}\right)\;+\;\eta_{\text{BSV}}\;+\;\eta_{\text{BOV}}\;+\;\varepsilon$ ${\Delta \text{QTcF}}_{\text{MFX/CFZ}}\;=\;\theta_{\text{TYP}}\;\times \;\left(\text{1}\;+\;\theta_{\text{CFZ+MFX}}\right)\;+\;\theta_{\text{age}}\;\times \;\left(\text{age}\;-\;2.83\right)\;+\;\theta_{{\text{QTcF}}_{0}}\;\times \;\left(\frac{{\text{QTcF}}_{\text{0}}\;-\;\text{363}}{\text{10}}\right)\;+{\text{η}}_{\text{BSV}}\;+{\text{η}}_{\text{BOV}}\;+\;\varepsilon$

Analysis of maximum absolute QTcF (QTcF_max_) revealed results similar to those for ΔQTcF. CFZ+MFX was significantly associated with a 13.0-ms increase in QTcF_max_ (ΔOFV = −5.733, *P* = 0.0166) in multivariate analysis after controlling for age (Table 4). BDQ use was associated with a 23.1-ms increase in QTcF_max_ (ΔOFV = −9.14, *P* = 0.0025) in the univariate analysis but was not significant in the multivariate analysis after controlling for age (Table S4). Time after dose at QTcF_max_, drug dose, or gender was not significant in univariate analysis (Table S4).

**TABLE 4:** Final model of the maximum absolute QTcF^*a*^

Parameter	Value (% RSE)
Typical QTcF_max_, θ_TYP_ (ms)	380 (0.7)
Effect of taking both clofazimine and moxifloxacin on QTcF_max_, θ_CFZ+MFX_ (ms)	13.0 (26.9)
Effect of age on QTcF_max_, θ_age_ (ms/yr)	3.8 (12.8)
Between-subject variability, η_BSV_ (ms)	18.5 (11.1)
Residual additive error, ε (ms)	14.2 (8.7)

a Abbreviations: QTcF, QT interval corrected by Fridericia formula; QTcF_max_, maximum absolute QTcF after drug administration. The final model described levofloxacin-based regimens (QTcF_max,LFX_) and moxifloxacin- or clofazimine-based regimens (QTcF_max,MFX/CFZ_) separately and was a function of age (θ_age_) and concomitant drug use: ${\text{QTcF}}_{\text{max},\text{LFX}}\;=\theta_{\text{TYP}}\;+\;\theta_{\text{age}}\;\times \;\left(\text{age}\;-\;2.83\right)\;+\;\eta_{\text{BSV}}\;+\text{ε}$ ${\text{QTcF}}_{\text{max},\text{MFX/CFZ}}\;=\theta_{\text{TYP}}\left(1\;+\;\theta_{\text{CFZ/MFX}}\right)\;+\;\theta_{\text{age}}\;\times \;\left(\text{age}\;-\;2.83\right)\;+\;\eta_{\text{BSV}}\;+\text{ε}$

## DISCUSSION

In this analysis of data from children aged <18 years treated for RR-TB in South Africa, we show that the risk of severe QTcF interval prolongation is low at the doses used in these studies, even when multiple QT-prolonging drugs are used concomitantly. No severe QT interval prolongation (>500 ms) occurred, and only a small proportion of children (7 of 88) experienced mild to moderate QT interval prolongation, most of whom were taking more than one QT-prolonging drug. To our knowledge, this is the first study to characterize QT interval prolongation in children with TB treated with standard-of-care regimens that include up to three QT-prolonging agents.

The use of multiple QT-prolonging agents is known to increase the risk of QT interval prolongation and adverse events (19). In analysis of ΔQTcF and QTcF_max_, we found that the risk of QT interval prolongation was highest with MFX+CFZ, while CFZ+BDQ or CFZ+LFX was not significantly different from CFZ alone. Our findings support a more-than-additive effect of CFZ+MFX on ΔQTcF in both univariate and multivariate analysis. On average, children in the CFZ+MFX group were receiving CFZ for a longer period prior to QTcF measurement than the CFZ-alone group. Given CFZ’s long elimination half-life of 10 to 12 days (25), CFZ accumulation may partly explain this finding. Yoon et al. found that more QTcF interval prolongation occurred in patients receiving CFZ and MFX than in those receiving the single drug (26). Up to a 2.5-fold-greater ΔQTcF has been reported in adults receiving CFZ with BDQ than in those receiving CFZ alone (15, 18, 27). Together, these findings suggest that use of CFZ with another QT-prolonging agent may result in more than a proportional ΔQTcF. Our analysis supports this for CFZ+MFX but not for CFZ+BDQ. Further investigation of QTcF interval prolongation with typical combination regimens for RR-TB treatment in children is required.

QT prolongation is concentration dependent, typically described through a linear concentration–QTc relationship (27–31). The daily MFX dose was significantly associated with an increase in ΔQTcF in univariate analysis but not in the multivariate model. In adults, MFX is associated with a 2- to 4.5-ms change in QTcF per mg/L (28), and more events of a QTcF being >500 ms occurred with 800 mg/day than with 400 mg/day in MDR-TB treatment (17). A study in children found a smaller effect of MFX concentration on QTcF in children with TB, but MFX concentrations overall were lower than those in adults (10). The CFZ concentration-QTcF relationship has been characterized in adults (32); however, studies in children are lacking. Future studies assessing ΔQTcF with respect to CFZ drug concentration in children are needed to fully understand how dose impacts QT prolongation risk.

In univariate analysis, the effect of CFZ alone was small and not statistically significant (mean ΔQTcF = −1.1 ms). A previous study in patients with nontuberculosis mycobacterial infection treated with CFZ found no significant QT prolongation; however, this study enrolled only 18 adult participants (33). Pym et al. reported an increase in grade 3 adverse events (QTcF of >500 ms) in patients taking CFZ (15). True baseline QTcF measures were not available in this study (i.e., prior to treatment initiation). Given CFZ’s pharmacokinetic properties and long elimination half-life, CFZ likely prolongs the QT interval over a duration longer than a single dosing interval. Evaluating QT interval changes from first dose to steady state (30 to 50 days) (25) will be valuable to understand true QT prolongation risk with CFZ use in children with TB who are treated with CFZ for several months.

Time after dose was not a significant factor in our analysis. This may be due to differences in peak concentration time between drugs. MFX concentrations peak between 2 and 4 h after dose (34), coinciding with the maximum ΔQTcF (35–38). CFZ concentrations peak at 4 to 8 h after dose (25) while the maximum concentration of BDQ is reached at 5 to 6 h after dose (39). In our study, the maximum ΔQTcF in MFX-treated children occurred at 3.6 h postdose versus 1.2 h postdose for CFZ-treated children. Mismatched peak ΔQTcF and concentration in CFZ-treated children may be due to saturation of hERG potassium channels (40, 41), given substantial CFZ drug accumulation at the time of QTcF sampling (46 days after CFZ initiation on average).

BDQ has been associated with QT prolongation in children and adults (42, 43). Greater QT prolongation was observed in adults when BDQ was used in combination with CFZ or MFX (15–21), along with more cases of severe QT prolongation and death (19). In our analysis, only 19 patients received BDQ, and all uses were in combination with another QT-prolonging agent (CFZ, LFX, or DLM). We found higher QTcF_max_ values in children with BDQ, but this effect did not retain statistical significance in multivariate analysis. This could be due to limited sample size, since only 13 of the 19 children were in the maintenance phase, when drug accumulation would be greatest. Since current WHO guidelines for the treatment of MDR-TB recommend a regimen containing a fluoroquinolone, BDQ, and CFZ, a complete understanding of BDQ’s QT-prolonging effects when used alone and in combination with CFZ and/or MFX/LFX is necessary in children to ensure safe use. Studies should evaluate longitudinal changes in QTcF from treatment initiation to completion and during the dosing interval given BDQ’s long elimination half-life (42, 43).

This study has some limitations. First, true baseline QTcF (prior to the initiation of RR-TB treatment) was not measured in the studies. Instead, the predose QTcF measure was used as the reference for ΔQTcF, and an analysis of the absolute QTcF_max_ was also performed. This study did not consider drug concentrations, so no conclusions can be drawn regarding dosing for combination regimens with increased QT prolongation risk. Further, sample size did not permit assessment of QT-prolonging effects with DLM alone or in combination with other drugs.

In conclusion, among children with RR-TB receiving one or more QT-prolonging drugs, the use of MFX and CFZ together had a greater effect on the QTcF interval than did MFX alone and CFZ alone. Characterizing concentration-QTcF relationships, especially for CFZ and MFX, will help to inform safe and effective doses for multidrug regimens for RR-TB that include multiple QT-prolonging agents. Understanding the longitudinal change in QTcF intervals with CFZ and BDQ use with or without other QT-prolonging agents is also needed.

## MATERIALS AND METHODS

### Study design and participants.

These data were collected from two prospective observational studies (MDRPK1, 2011 to 2015; MDRPK2, 2016 to 2020) and include drugs used, safety, and treatment outcomes of the second-line TB drugs in children with RR-TB in the Western Cape, South Africa. HIV-positive and HIV-negative children aged <18 years with probable or confirmed RR-TB were enrolled. Children enrolled in both studies were routinely treated with second-line anti-TB drugs based on national treatment guidelines at the time of the studies. Throughout both study periods, local guidelines recommended the use of MFX in children ≥8 years of age and LFX for children <8 years due to lack of child-friendly drug formulations. Initially, CFZ was reserved for treatment of MDR-TB, i.e., RR-TB with resistance to fluoroquinolones and/or second-line injectable agents; in 2016, CFZ was permitted in RR-TB treatment regimens, in accordance with national and international guidelines. BDQ became accessible through the national tuberculosis program in 2015. Access to DLM remained limited throughout the study period, but from 2017 was available through a national clinical access program for patients with limited treatment options. Patients were receiving routine MDR/RR-TB treatment for at least 2 weeks and <16 weeks at enrollment. Study details have been described previously (10, 44).

### QT interval assessment.

A 12-lead electrocardiogram (ECG) was performed in triplicate before observed drug administration (predose) and at 1, 4, and 10 h after dose (MDRPK2) or at 2 h after dose (MDRPK1). QT intervals were corrected for heart rate using the Fridericia correction formula (QTcF) (45), and the mean of triplicate QTcF measures was calculated. QTcF prolongation was graded as follows using the mean of the triplicate: grade 1 (mild), QTcF interval >450 to ≤480 ms; grade 2 (moderate), QTcF interval >480 to ≤500 ms; grade 3 (severe), QTcF interval >500 ms (45). The change in QTcF of the mean of the triplicate from the predose measure (ΔQTcF) was calculated.

### Statistical analysis.

Statistical analysis was done using STATA (version 15; StataCorp, College Station, TX, USA), R Statistical Software (version 3.4.3; https://www.r-project.org/), and NONMEM software (version 7.4; Icon Development Solutions, Ellicott City, MD). Data visualization was performed using R Statistical Software (version 3.4.3; https://www.r-project.org/). Age, weight, and height measurements were used to calculate weight-for-age z-score (WAZ), height-for-age z-score (HAZ), and weight-for-height z-score (WHZ) using the WHO Anthro macro for Stata (46–48). A child was considered underweight, stunted, or wasted if the child had a WAZ of <−2, a HAZ of <−2, or a WHZ of <−2, respectively. CFZ and BDQ doses were summarized as weekly dose to normalize between children requiring different dosing frequencies according to weight. For summary of adverse events, children who received LFX alone were used as the reference since LFX-induced QT prolongation is minimal (49–52).

### (i) Linear models.

A linear regression analysis was conducted on the maximum ΔQTcF. Each patient contributed one value, which was the maximum ΔQTcF over all patient-visits. Covariates evaluated included drug regimen, gender, age, and predose QTcF (QTcF_0_).

### (ii) Linear mixed-effects model.

To quantify the effect of potential drug interactions on the ΔQTcF and maximum absolute QTcF (QTcF_max_), linear mixed-effects analysis was performed with NONMEM. Variability was considered additive, including for between-subject variability (BSV), between-occasion variability (BOV), and unexplained residual variability. Nested models were assessed by their objective function value (OFV), computed by NONMEM to be proportional to −2 times the log likelihood. At a *P* value of 0.05, a decrease in the OFV of at least 3.84 points was considered a statistically significant difference when comparing two models with one parameter difference (χ^2^ distribution with 1 degree of freedom [df]). ΔQTcF and QTcF_max_ were modeled according to equation 1, where the intercept represents the typical ΔQTcF or QTcF_max_ in the population, η is the combined BSV and BOV, and ε is the residual variability. Since LFX and MFX were never used together, two intercepts were estimated *a priori*: one for children receiving an LFX-based regimen and the other for children not receiving an LFX-based regimen. As part of the MDRPK2 study design, some children switched from LFX to MFX during the study. Thus, potential differences in ΔQTcF within a patient who switched regimens were captured with different intercepts as well as BOV. The factors evaluated for their effect (EFF) on ΔQTcF or QTcF_max_ were second-line MDR-TB drug used (CFZ, MFX, BDQ, and DLM) given alone or in combination, drug dose, time after dose, weight, QTcF_0_, age, and sex. Linear and exponential relationships were tested for continuous covariates and additive relationships were tested for categorical covariates by using a stepwise forward inclusion (*P* < 0.05) and backward elimination (*P* < 0.01) approach (53).

(1) $$\Delta \text{QTcF}\;\text{or}\;{\text{QTcF}}_{\text{max}}\;=\;\left(\text{intercept}+\text{η}\right)\;+\;{\text{EFF}}_{1}\;+\;{\text{EFF}}_{2}\;+\;{\text{EFF}}_{3}\;+\;\text{…}\;+\text{ε}$$

### Ethics.

Written informed consent was provided by the parent(s) or legal guardians, and children of ≥7 years of age provided written informed assent. The studies were approved by the Health Research Ethics Committee of Stellenbosch University (N11/03/059, MDRPK1; N15/02/012, MDRPK2) and the local health departments and hospitals.
