# Supplementary material for: QT Interval Prolongation with One or More QT-Prolonging Agents Used as Part of a Multidrug Regimen for Rifampicin-Resistant Tuberculosis Treatment: Findings from Two Pediatric Studies
Source: Antimicrob Agents Chemother. 2023 Jun 26;67(7):e01448-22. doi: 10.1128/aac.01448-22 (PMC10353402; doi:10.1128/aac.01448-22)
Supplement: Supplemental file 1 — Supplemental material. Download aac.01448-22-s0001.docx, DOCX file, 0.02 MB [file aac.01448-22-s0001.docx]

**Supplemental material**

| **Table S1. Duration of CFZ at each pharmacokinetic visit** | | |
| --- | --- | --- |
|  | ECG visit 1 (n = 63) | ECG visit 2 (n = 50) |
| CFZ alone | 36 (14 , 132) | 52** |
| CFZ + LFX | 39 (8 , 118) | 57 (18 , 107) |
| CFZ + MFX | 57 (41 , 112) | 77 (22 , 127) |
| CFZ + BDQ | 64 (62 , 78) | 86* |
| CFZ + LFX + BDQ | 28 (8 , 121) | 34 (15 , 162) |

*Based on two patients; **Based on one patient; values are reported as median (2.5th , 97.5th range) days; PK, pharmacokinetic; LFX, levofloxacin; CFZ, clofazimine; MFX, moxifloxacin; BDQ, bedaquiline.

| **Table S2. Linear Regression of maximum ∆QTcF (ms)** | | | | | |
| --- | --- | --- | --- | --- | --- |
| **Parameter** | **Univariate analysis** | |  | **Multivariate analysis** | |
|  | **Estimate (95% CI)** | **p-value** |  | **Estimate (95% CI)** | **p-value** |
| $\epsilon$ , Intercept (ms) | - | - |  | 14.5 (10.1, 18.9) | <0.001 |
| $\theta$, QTcF_0_ (ms/10 ms) | - | - |  | -2.1 (-3.3, -0.9) | 0.001 |
| $\theta$, LFX | -1.4 (-8.3, 5.6) | 0.694 |  | - |  |
| $\theta$, CFZ | 4.5 (-2.9, 11.9) | 0.228 |  | - |  |
| $\theta$, MFX | 2.5 (-4.4, 9.4) | 0.471 |  | - |  |
| $\theta$, CFZ+MFX | 10.5 (3.3, 17.8) | 0.005 |  | 13.4 (6.1, 20.7) | <0.001 |
| $\theta$, CFZ+LFX | -4.1 (-12.9, 4.7) | 0.353 |  | - |  |
| $\theta$, CFZ+LFX+BDQ | -3.8 (-14.5, 6.8) | 0.476 |  | - |  |
| $\theta$, Gender | 0.4 (-6.4, 7.2) | 0.913 |  | - |  |
| $\theta$, Age (ms per year) | 0.7 (-0.1, 1.5) | 0.102 |  | 1.1 (0.3, 1.9) | 0.007 |
| $\theta$, Weight (ms per kg) | 0.2 (-0.1, 0.6) | 0.173 |  | - |  |

∆QTcF, change in QTcF (ms) after dose; CI, confidence interval; CFZ, clofazimine; MFX, moxifloxacin; LFX, levofloxacin; BDQ, bedaquiline; ${QTcF}_{0},$ QTcF at pre-dose (or time after dose = 0).

In the multivariate model, maximum ∆QTcF after dose is a function of ${QTcF}_{0}$, Age, and CFZ+MFX (1: yes, 0: no) expressed as:

$\theta_{\mathrm{QTcF}0}\times\frac{{QTcF}_{0}-363}{10}+ \theta_{CFZ+MFX}\times\left( CFZ+MFX \right)+ \theta_{Age}\times\left( Age-2.83 \right)+ \epsilon$

| **Table S3A. Summary of the change in QTcF (∆QTcF) after dose** | | | | |  |
| --- | --- | --- | --- | --- | --- |
|  | Received at ECG visit | | p* |  | |
|  | Yes | No |  |  | |
| LFX | -0.61 | 4.47 | 0.0607 |  | |
| CFZ | 5.46 | 3.77 | > 0.05 |  | |
| MFX | 7.02 | 3.11 | > 0.05 |  | |
| CFZ+LFX | -2.58 | 5.96 | 0.0002 |  | |
| CFZ+MFX | 11.63 | 1.76 | < 0.0001 |  | |
| CFZ+BDQ | -1.36 | 3.98 | > 0.05 |  | |

Values reflect the mean ∆QTcF for each drug at a particular visit. A child can appear on more than one drug depending on visit. ∆QTcF, change of QTcF (ms) at time after dose from pre-dose; QTcF, QT interval corrected by Frederica formula. *P-values were obtained after fitting ∆QTcF with each of the covariate controlling for pre-dose QTcF and between subjects and occasion (visit) variabilities.

| **Table S3B. Univariate analysis of the change in QTcF (∆QTcF) after dose** | |
| --- | --- |
| **Parameter** | **Value** |
| CFZ dose per week, (ms/mg/kg) | -0.005 (ns) |
| MFX dose per day, (ms/mg/kg) | 0.6* |
| LFX dose per day, (ms/mg/kg) | -0.4* |
| Time after dose, (ms/hour) | 0.03 (ns) |
| Female gender | 4.5 (ns) |
| Age, (ms/year) | 0.9* |
| Weight, (ms/kg) | 0.4* |

Values reflect the mean-change in ∆QTcF for each covariate. *, p<0.05; ns, not statistically significant; ∆QTcF, change in QTcF (ms) after dose; QTcF, QT interval corrected by Frederica formula. Estimates were obtained after controlling for pre-dose QTcF.

| **Table S4. Univariate analysis of maximum absolute QTcF (ms), QTcF_max_** | | |
| --- | --- | --- |
| **Parameter** | Value (% RSE) |  |
| Typical *QTcF_max_* in participants receiving LFX, (ms) | 390 (0.9) |  |
| Typical *QTcF_max_* in participants receiving MFX or CFZ without LFX, (ms) | 397 (0.8)* |  |
| Covariate effects on *QTcF_max:_* |  |  |
| CFZ+LFX*,* (ms) | -9.8 (48.8)* |  |
| CFZ+MFX*,* (ms) | 7.7 (65.6) |  |
| CFZ+BDQ*,* (ms) | 18.5 (91.9) |  |
| BDQ*,* (ms) | 23.1 (32.2)* |  |
| CFZ+LFX+BDQ*,* (ms) | 13.8 (58.9) |  |
| CFZ dose per week, (ms/mg/kg) | -0.1 (101.5) |  |
| MFX dose per day, (ms/mg/kg) | -0.3 (210.5) |  |
| LFX dose per day, (ms/mg/kg) | -0.3 (249.5) |  |
| Time after dose (ms/hour) | 0.6 (76.5) |  |
| Female gender | 8.8 (68.3) |  |
| Age (ms/year) | 3.7 (13.5)* |  |
| *Statistically significant at 5% significant level.  CFZ, clofazimine; MFX, moxifloxacin; LFX, levofloxacin; BDQ, bedaquiline; | | |
